# Editorial: Recent Trends in Integrated Wastewater Treatment for Sustainable Development

**DOI:** 10.3389/fmicb.2022.846503

**Published:** 2022-03-31

**Authors:** Vineet Kumar, Muhammad Bilal, Luiz Fernando Romanholo Ferreira

**Affiliations:** ^1^Waste Re-processing Division, CSIR-National Environmental Engineering Research Institute (CSIR-NEERI), Nagpur, India; ^2^School of Life Science and Food Engineering, Huaiyin Institute of Technology, Huaian, China; ^3^Waste and Effluent Treatment Laboratory, Institute of Technology and Research (ITP), Tiradentes University, Aracaju, Brazil; ^4^Graduate Program in Process Engineering, Tiradentes University, Aracaju, Brazil

**Keywords:** phytotreatment, refractory contaminants, tertiary treatment, bioaugmentation, pulp-paper industry

Water, one of the utmost valuable natural resources for life on Earth, is critical to our society's economic development and success (agriculture, industry, and energy production). Nothing is more important to life on Earth than water. Although water is abundant on Earth, covering around 70% of the surface area, only 1% of that resource is available for human consumption. Water usage is significantly increasing in many parts of the world as a result of rising population, increasing economic activities, widespread industrialization, and urbanization, which are all raising living standards and boosting food demand. Thus, the problem of freshwater scarcity and contamination of natural water resources has become a menace to the developing globe that requires rapid response (Yaashikaa et al., 2022). Worldwide the freshwater use is projected to have increased twice as quickly as the human population. In 2050, consumption is predicted to be tremendously higher than it is today, with over 40% of the world's population facing water scarcity issues. These factors compel us to consider non-conventional water sources as a means of meeting the growing need for fresh water. Wastewater and waste treatment are feasible options for dealing with freshwater scarcity. Various industries, municipalities, agricultural lands, commercial regions, and urban areas all emit large amounts of highly contaminated wastewater (Nookwam et al., 2022). According to the World Health Organization (WHO), providing good quality water for sustainable development of the society, wastewater treatment is essential before its discharge into surface water systems to protect the environment and public health.

According to Lamastra et al. (2016), more than 700 different metabolites and emerging pollutants are discharged into the environment without treatment. This fact is further worse by the lack of knowledge about the transportation, destination, and toxic potential of these toxic contaminants when they entered into the ecosystem, besides hampering the development of environmental policies controlled by regulatory agencies that promote the sustainable treatment and management of pollutants in the open environment (Taheran et al., 2018). However, the persistent increase of these in the contaminated site still present due to the consumerist profile of the current society (Vasilachi et al., 2021). In this context, biocatalysis plays a vital role in the growth of many industries, such as food, energy, and fine chemistry, since this green alternative allows replacing traditional processes through the use of microorganisms and their enzymes in catalytic reactions that can be applied in the biodegradation of various organic and emerging contaminants present in various agro-industrial and industrial effluents, reducing environmental concerns and improving the water quality after the use of microbiotecnologies.

Physicochemical approaches such as membrane filtration, ion exchange, advanced oxidation processes, and coagulation/flocculation, despite their efficacy, are not preferred due to sensitivity to variable water input, excessive chemical consumption, high operational costs, and the generation of a large amount of sludge as well as secondary pollutants. Although traditional wastewater treatment technologies are widely used, they fail to eradicate numerous complex contaminants. These chemicals eventually make their way into wastewater, posing a threat to water quality (Fito and Van Hulle, 2021; Daud et al., 2022). Even after secondary treatment, wastewater is expected to produce substantial volumes of new and complex contaminants that are always more difficult to deal with. Adequate wastewater treatment is rarely achieved by the use of a single treatment technique.

There is an urgent need to develop a cost-effective, efficient, and comprehensive technologies or improve the existing methods through some interventions for adequate treatment and eliminating pollutants from wastewater to achieve environmental sustainability (Varjani et al., 2020; Shah et al., 2022). In the recent past, innovative integrated treatment technologies by coupling two or more biological, physical, and chemical processes to remediate or clean up many environmental contaminants from wastewater have been gained worldwide attention (Figure 1). Overall, this Research Topic (RT) provided an appropriate platform for discussing current trends, developments, and applications of integrated treatment technologies in the degradation and detoxification of wastewater or pollutants, including new emerging contaminants, industrial discharges, and municipal wastewater treatment plants.

**Figure 1:**
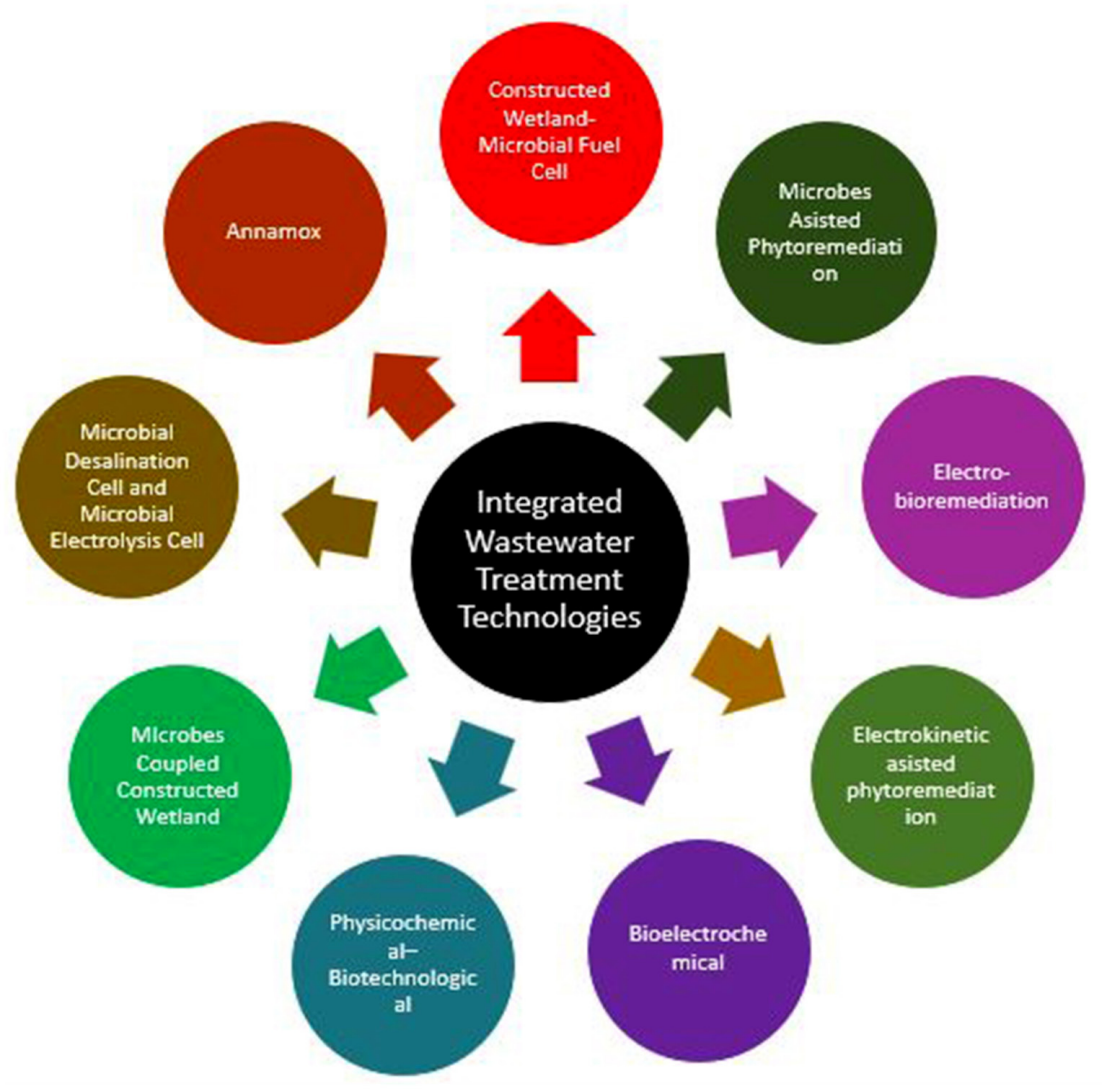
Various integrated environmental technology used for wastewater treatment and management.

This RT consists of three research articles and four reviews contributed by leading experts from around the globe. The first research article, which was contributed by Verdel et al. and the team, has focused on the development of a biobased approach for the remediation of white water utilizing an indigenous bacteria isolate. Authors developed a bacterial consortium comprises *Aeromonas* sp. RES19-BTP, *Cellulosimicrobium* sp. AKD4- BF, *Sphingomonas* sp. BLA14-CF, and *Xanthomonadales* sp. CST37-CF for degradation of organic additives. The consortium immobilized on carriers showed a reduction of COD up to 88% in the white-water in a pilot-scale 33-liter tubular reactor filled with white water even after 21 days. Marathe et al. investigated the phytotreatment efficiency of low-cost organic raw materials for the remediation of saline effluent generated by tannery, textile, and pulp-paper industries. According to the Food and Agriculture Organization (FAO), wastewater having high total dissolved solids (TDS; 2,000 mg/L) is laid under severe restriction use for irrigation due to high salt accumulation in crop roots resulting in a total loss in crop yield. Authors used saline wastewater had high TDS (6,143.33 mg/L) for its treatment using lysimeters aided with organic filter bedding raw material as planted with potential plant, *Eucalyptus camaldulensis*. *E. camaldulensis* plant species displayed high efficiency to treat wastewater simultaneous accumulation of the high amount of sodium, calcium, magnesium, potassium, and produce higher biomass, and chlorophyll content without showing any negative impact on the growth as a function of wastewater. The high TDS management efficiency of coconut and rice husk as bedding material in a combination with forestry plant species was highest compared to other tested organic raw materials. The authors concluded that organic waste based amendments are an eco-sustainable option for managing saline wastewater.

Mishra et al. reviewed bacterial-assisted degradation of toxic dyes used in textile, cosmetics, and pharmaceuticals industries and other co-contaminants that pose a significant threat to humans and the ecosystem. Thus, public demands for decontaminated effluent to the receiving environment have made degradation, decolorization, and detoxification of contaminated wastewater discharges from industries a top priority. The authors highlight the decolorization and biodegradation mechanisms of various azo dyes by several bacterial enzymes. In addition, molecular docking of bacterial enzymes with dye molecules gives a new insight at the molecular level to explore the detailed mechanism of bacterial enzymes in dye degradation. The authors also discussed the molecular and physiological capabilities of isolates, which were beneficial for bioremediation processes.

Mathew et al. summarized the integrated algae and bacterial approaches for the degradation of contaminated sewage water. Due to the presence of an overwhelming blend of numerous refractory chemicals, the conventional approaches are unsuitable neither as on-site nor as a centralized treatment, and the urgent need of specific treatments is required. The integrated use of microbes for biodegradation is convenient because it is versatile, has dynamic metabolisms. The direct application of algae also helps to remove toxic heavy metals like aluminum, nickel, copper, etc. with simultaneous reduction of nutrients from the wastewater. Their growth also creates the potential for biofuels and bioproducts from their biomass. The augmentation of potent bacterial islates further enhanced the remediation efficacy of the wastewater. This happens as the inorganic nutrients was assimilated into the algal biomass whereas bacteria utilized organic nutrients. Furthermore, the mutualistic exchange of carbon dioxide and oxygen between bacterial and algae species helps intensify the photosynthetic activity of algae while oxidation-reduction by bacteria lead to the removal of nutrients from wastewater.

In general, one of the key motives of research organizations around the world is to develop technological improvements directed at the production chain of sustainable processes. Beyond the current topic's scientific, technological, and environmental importance, it is critical to develop integrated treatment methods for long-term development, using alternate reuse techniques concurrently to treat these residues and other resistant harmful chemicals. The latest recent innovations in integrated wastewater treatment methods provide an option to deal with organic and developing pollutants and increase process efficiency, making biotech and associated businesses more appealing because they use higher added value renewable inputs. At the same time, it enables creative methods to treat these residues and other resistant aromatic compounds via biological treatment systems, with the possibility of linking to new processes, and the practicality of deployment in biorefineries and meet the circular bioeconomy.

In summary, the new knowledge provided by the articles in this RT will expand our understanding of the current development in wastewater treatment by emerging integrated approaches and enable us to reach eco-sustainable treatment technology to obtain safe wastewater.

## Author Contributions

VK drafted this editorial, which all authors reviewed and commented on, and eventually approved the final version of the manuscript for publication.

## Conflict of Interest

The authors declare that the research was conducted in the absence of any commercial or financial relationships that could be construed as a potential conflict of interest.

## Publisher's Note

All claims expressed in this article are solely those of the authors and do not necessarily represent those of their affiliated organizations, or those of the publisher, the editors and the reviewers. Any product that may be evaluated in this article, or claim that may be made by its manufacturer, is not guaranteed or endorsed by the publisher.
